# Toward Clinically Compatible Phase-Contrast Mammography

**DOI:** 10.1371/journal.pone.0130776

**Published:** 2015-06-25

**Authors:** Kai Scherer, Konstantin Willer, Lukas Gromann, Lorenz Birnbacher, Eva Braig, Susanne Grandl, Anikó Sztrókay-Gaul, Julia Herzen, Doris Mayr, Karin Hellerhoff, Franz Pfeiffer

**Affiliations:** 1 Lehrstuhl für Biomedizinische Physik, Physik-Department & Institut für Medizintechnik, Technische Universität München, Munich, Germany; 2 Institute of Clinical Radiology, Ludwig-Maximilians-Universität München, Munich, Germany; 3 Institute of Pathology, Ludwig-Maximilians-Universität München, Munich, Germany; Illinois Institute of Technology College of Science, UNITED STATES

## Abstract

Phase-contrast mammography using laboratory X-ray sources is a promising approach to overcome the relatively low sensitivity and specificity of clinical, absorption-based screening. Current research is mostly centered on identifying potential diagnostic benefits arising from phase-contrast and dark-field mammography and benchmarking the latter with conventional state-of-the-art imaging methods. So far, little effort has been made to adjust this novel imaging technique to clinical needs. In this article, we address the key points for a successful implementation to a clinical routine in the near future and present the very first dose-compatible and rapid scan-time phase-contrast mammograms of both a freshly dissected, cancer-bearing mastectomy specimen and a mammographic accreditation phantom.

## Introduction

With the introduction of a three-grating Talbot-Lau interferometer using structured gratings, phase-contrast imaging is no longer restricted to highly brilliant X-ray sources, but instead is compatible with conventional laboratory sources, so that great potential for clinical applications is envisioned [1]. Especially in the case of mammography, recent studies verified diagnostic advantages for certain mammographic indications, when utilizing phase- and scatter-sensitive imaging [2, 3, 4 and 5]. However, a major shortcoming of all previous studies on laboratory phase-contrast mammography was that the underlying image acquisition parameters did not meet clinical standards with respect to radiation dose, scan-time and consistency between subsequent measurements. While Scherer *et al*. could obtain comprehensive phase-contrast mammograms of fresh and un-fixated, cancerous mastectomy specimen at a medical sample compression using a compact setup (total length of 1.5m, rotating X-ray anode, flat panel x-ray detector with 127 x 127 um^2^ pixel size, effective pixel size of 85 x 85 um^2^—similar in its design to a conventional mammography systems) the mean glandular dose (MGD) of 66 mGy per measurement by far exceeded the dose limits of 2.5 mGy, set by the European guidelines for Quality assurance in Mammography [2,6]. Also, the exposure times used for this initial proof-of-principle study amounted to more than one minute, incompatible with clinical routine and patient care. Similar limitations (MGD of 26 mGy, exposure time of 72 seconds) have been reported by other groups working in the field of grating-based phase-contrast mammography [3, 4].

Therefore, a two-fold problem, strongly restricting the further progression of phase-contrast mammography towards clinical implementation, arises. Firstly, high-dose measurements may not be reproducible within a dose-compatible scenario, which weakens the diagnostic significance and transferability of current studies. Secondly, the analyzed sample collective is limited to a small number of ex-vivo mastectomy samples comprising medical indications which may not be representative for daily screening. To overcome these limitations and to further pave the way of phase-contrast mammography into daily, clinical routine, we present the very first dose-compatible breast and phantom measurements scanned within only 12 seconds, while providing image quality and acutance equivalent to clinical mammograms.

## Materials and Methods

The study was conducted in accordance with the Declaration of Helsinki and was approved by the local ethics committee (Ethikkommission of the Ludwig-Maximilian-University, Munich, project number 240–10, date of permission 26/08/2010, amendment 30/05/2012). Inclusion criteria were indication for surgical removal of a benign or malignant breast tumor after previous core biopsy. Participants gave written consent before participation after adequate explanation of the study protocol. Indication for breast surgery followed recommendation of the interdisciplinary tumor board of the University of Munich breast center.

### Setup Optimization

**Fig 1:**
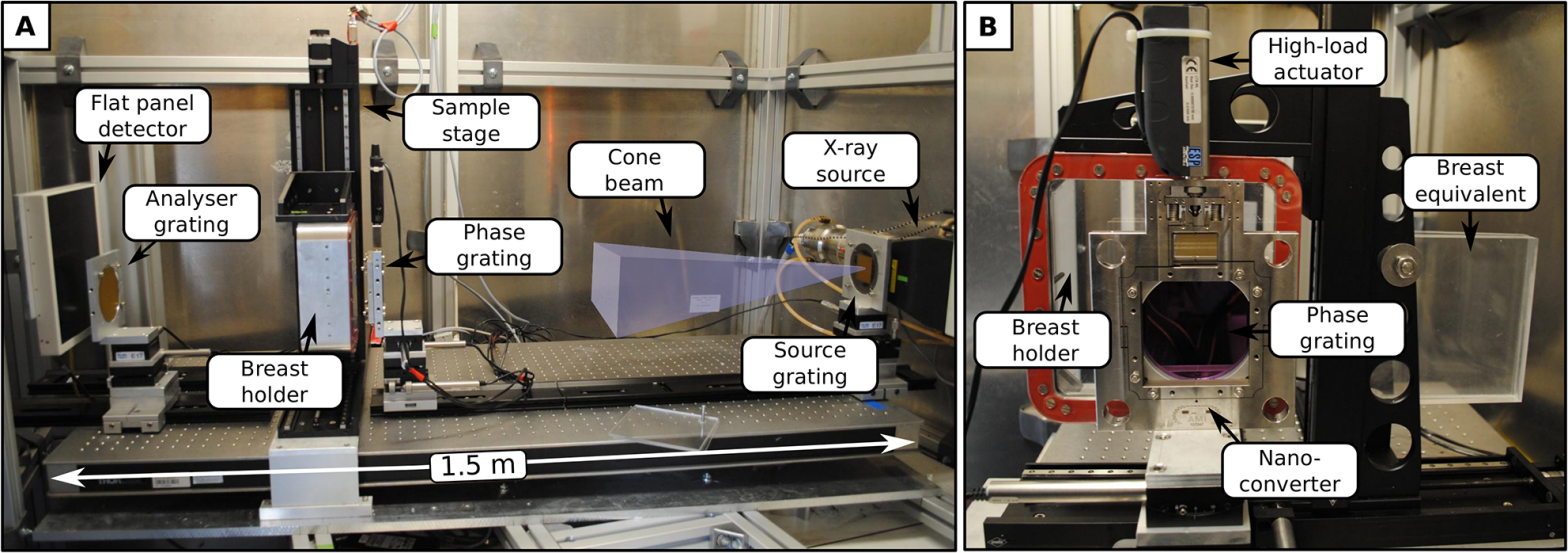
Laboratory X-ray phase-contrast mammography setup. (A) Longitudinal view of the revised, laboratory X-ray phase-contrast mammography setup with a dose-saving arrangement of phase grating and sample holder. A compact system length, rotating X-ray anode and conventional flat panel detector met the design criteria of clinical mammography systems. (B) Close-up view in the direction of the X-ray beam showing the phase-stepping instruments and breast equivalent incorporated for flat-field measurements. The combination of high-load actuator and nano-converter enables image acquisition times within 12 seconds.

Several software and hardware specifications of the dedicated phase-contrast mammography system (Fig 1A), which was used for previous studies, were optimized in order to enable clinically compatible measurements. The original values used for the previous high-dose measurements are listed in brackets–for more details see [2].

Firstly, the arrangement of the breast tissue holder and the phase grating downstream of the X-ray beam were exchanged [7]. Thus, a saving of approximately 33% mean glandular dose was achieved (value corresponds to a sample compression of 4 cm), by utilizing the wafer-substrate (silicon) as an effective absorber of low energy photons and increasing the source-to-sample distance (Fig 1A). Note that this optimization step only altered the effective pixel-size, whilst maintaining the overall photon statistics and only slightly decreasing the interferometer sensitivity, contingent on the fact that the sample holder was placed directly downstream of the phase grating (distance of 2.5 cm) [7].

Secondly, a Varian 2520-DX detector (Gadox-screen) with improved read out electronics and a 16-bit AD-converter was implemented as an integrating flat panel detector. Since the two absorber gratings reduce the total photon flux by at least 75%, the internal readout speed of the detector was strongly decreased to compensate for low photon statistics and avoid an accumulation of internal readout-noise during a single exposure. Correspondingly the factory setting of 10 fps (frames per second) was reduced to 1 fps and synchronized with the total exposure time of 1 (9) second per phase-step. To further decrease readout noise, radiation dose and scan-time, the number of phase-steps was decreased to 5 (9)—at which sufficient sampling of the phase-stepping curve is still practicable—resulting in a total exposure time of only 5 seconds (81) per measurement.

By tuning the acceleration speed of the high-load actuator, which translates the phase grating by means of a nano-converter, the scan time per projection and 5 phase-steps was lowered to 12 (101) seconds in total. Furthermore, the substrate thickness of the analyser grating was reduced to 200 (500) μm, in order to minimize the attenuation of dose-relevant photons.

Finally, a stack of Polymethylmethacrylat-plates (PMMA) of variable thickness was introduced into the flat-field measurement as a breast absorber equivalent (Fig 1B). Here, the breast equivalent serves multiple purposes: first, a saturation of the detector during flat-field measurements at low frame-rates is prevented. Secondly, the breast and respective reference scan exhibit comparable X-ray imaging spectra. As a consequence, shadowing artifacts appearing at the edges of the absorption gratings are eliminated; hence flat image backgrounds within the absorption and dark-field channel are provided, which strongly facilitate the depiction of fine and low-contrast features. Further, variations in the overall fringe visibility due to beam-hardening effects are avoided, preventing the generation of an artificial dark-field signal. Hence, by adapting the PMMA thickness so that the absorption power of the respective breast sample is mimicked for a certain effective energy, quantitativeness of each image signal is provided and therewith comparability between different breast samples established.

### Radiation Dose

In a first step, the incident air kerma was measured using an ionization chamber, which was positioned at the respective breast sample position. To approximate the mean glandular dose (MGD) deposited within the radiation sensitive glandular breast tissue, the incident air kerma (1.19mGy/s) was then multiplied with a breast-thickness dependent conversion factor g. The respective g-factors were calculated using Monte Carlo simulations (software package *CatSim*, GE Global Research), taking into account the setup-specific X-ray spectrum and beam filtration [8]. A standard breast phantom was used for the dose model and was simulated as a radiation sensitive tissue core (50% glandular and 50% adipose tissue) of variable thickness surrounded by a 5 mm thick layer of radiation-insensitive skin (100% adipose tissue). For this configuration, we obtained a half-layer value of 0.881 mm aluminum and g-factors ranging from 0.450 to 0.274 for 3 cm to 6 cm of breast tissue, respectively.

## Results

**Fig 2:**
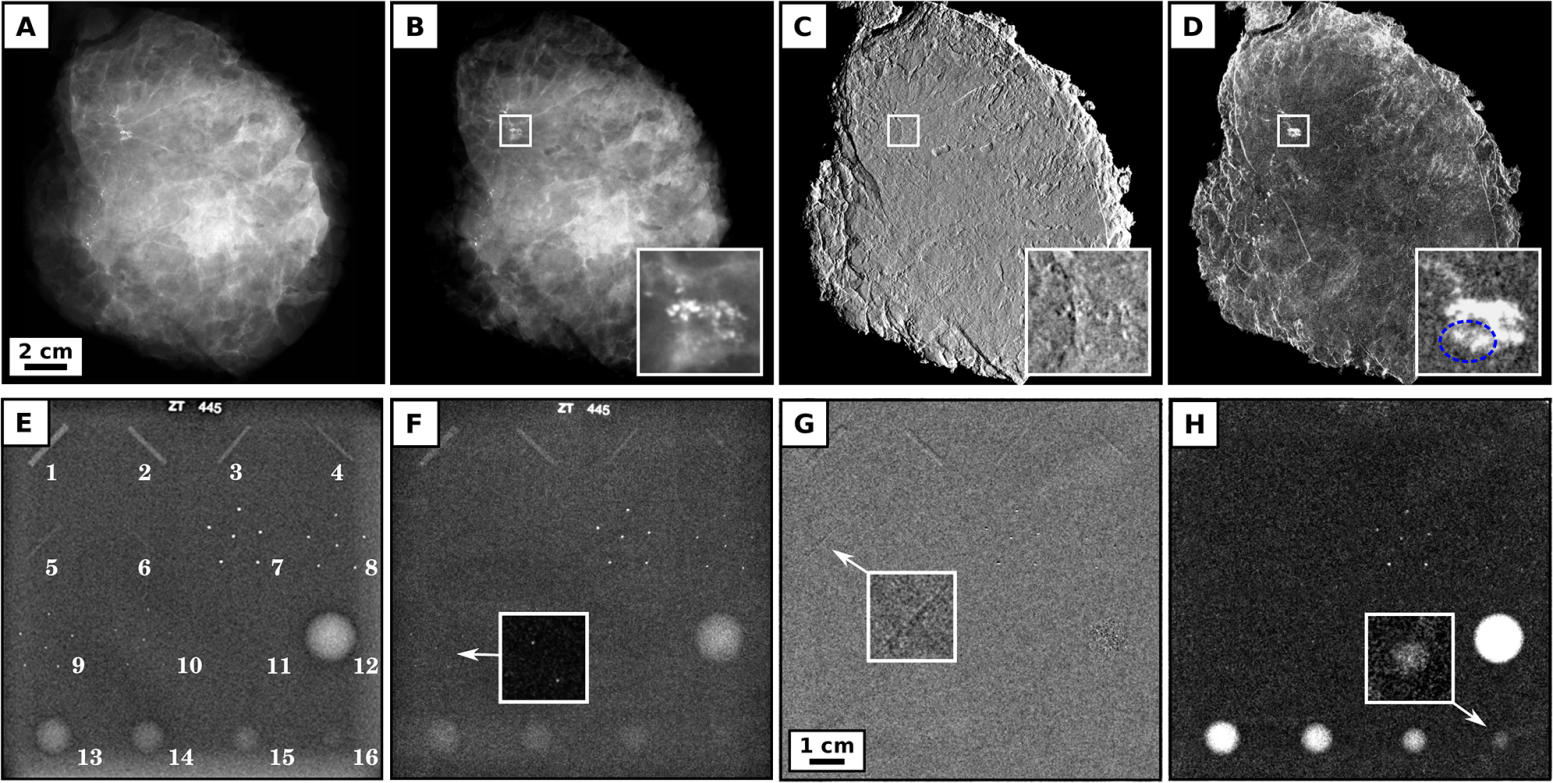
Clinically compatible phase-contrast mammograms of a freshly dissected, cancerous mastectomy sample and the mammographic accreditation phantom Gammex 156. Clinical ex-vivo mammography at 28 kVp, 86 mAs and 0.94 mGy mean glandular dose (Rhodium filter) (A), grating-based absorption (B), differential phase (C) and dark-field mammography (D) at 40 kVp, 70 mA and 2.2 mGy mean glandular dose of a freshly dissected mastectomy sample. Both absorption images are rated equivalent with respect to image quality and detection quality. Inlays show magnified view of the cancerous and micro-calcified tissue volume, with a superior contrast-to-noise ratio in the dark-field (10.6) in comparison to absorption channel (5.7). Notice that soft-tissue components of the tumor are exclusively detected within the dark-field signal, as indicated by the dashed blue line. Clinical mammography at 28 kVp, 159 mAs and 1.62 mGy mean glandular dose (Rhodium filter) (E), grating-based absorption (F), differential phase (G) and dark-field mammography (H) at 40 kVp, 70 mA and 2.07 mGy mean glandular dose of the mammographic accreditation phantom Gammex 156 (Gammex Inc., Middleton). The grating-based absorption image meets the standard criteria of clinical image quality, by resolving 4 of a minimum of 4 fibrils (#1–4), 3 of a minimum of 3 groups of simulated micro-calcifications (#7–9) and 4 of a minimum of 3 tumor masses (#12–15). The differential phase and dark-field channel provide complementary information by revealing an additional 5^th^ fibril (#5) and 5^th^ tumor mass (#16) as indicated by arrows.

The top row of Fig 2 shows the clinical mammogram of a freshly dissected, ex-vivo breast mastectomy sample (A) taken with a Hologic, Selenia Dimensions mammograph (Bedford, USA—pixel size of 70 x 70 μm^2^) and the corresponding grating-based absorption (B), differential phase (C) and dark-field mammogram (D), obtained at the laboratory X-ray phase-contrast mammography unit. The specimen was compressed to a thickness of 3.8 cm which corresponds to the clinical setting of a small breast. With a corresponding g-factor of 0.371 and a total exposure time of 5 seconds, the mean glandular dose was determined to be 2.2 mGy, which is approximately twice the dose of the respective conventional mammogram of 0.94 mGy, but below the guideline limit of 2.5 mGy [6]. Two experienced radiologists rated the clinical and grating-based absorption images as equivalent with respect to image quality, acutance, sharpness and depiction of tissue strands/microcalcifications. We conducted exemplary contrast-to-noise ratio (CNR) investigations within the cancerous and microcalcification bearing tissue volume (white framed inlay). We found that the dark-field channel yields a superior CNR of 10.6 in the depiction of the central tumor mass in comparison to 5.7 in the case of the absorption channel, proving that scatter-sensitive imaging provides enhanced detection sensitivity, even within a dedicated, low-dose setting. Of major diagnostic importance is further the observation that soft-tissue components of the tumor are exclusively resolved and clearly discriminated from surrounding tissue within the dark-field signal, as indicated by the dashed blue line. Here, scatter-sensitive imaging clearly outperforms conventional absorption-based mammography, since providing sub-resolution sensitivity towards highly dispersed calcium grains being indicative of malignancy in the reported case [5]. Histological workup of the mastectomy sample proved a residual (after neoadjuvant chemotherapy) breast cancer of non-specific type (NST) with a diameter of 11.5 x 8.5 x 2 cm as well as residual ductal carcinoma in situ containing intraluminal calcifications.

To ensure that low-dose, grating-based mammography meets the standards of clinical image quality, a mammographic accreditation phantom (Gammex 156, Gammex Inc., Middleton—4.2 cm compressed human breast, 50% adipose and 50% glandular tissue) was measured with the clinically compatible acquisition protocol (5 seconds exposure, 12 seconds scan time, MGD of 2.07 mGy). The respective absorption image (Fig 2F) even exceeds the criteria set by the State Departments of Radiologic Health and the ACR, by revealing 4 of a minimum of 4 fibrils, 3 of a minimum of 3 groups of simulated micro-calcifications and 4 of a minimum of 3 tumor masses [9]. Besides, the differential phase (G) and dark-field mammogram (H) enable the detection of a 5^th^ fibril and a 5^th^ tumor mass, as indicated by arrows, unseen within the absorption channel. Although the Gammex 156 can only reliably model the absorption behavior of breast features, the depiction of additional structures, proves complementarity of the three image modalities and shows that the strongly decreased number of phase steps and exposure time are still sufficiently high for a meaningful retrieval of (differential) phase and dark-field signal.

## Discussion

In this technical developments article, we have shown for the very first time that comprehensive phase-contrast mammography, using a compact laboratory setup, can be conducted in a clinically compatible manner. Our results demonstrate that the obtained absorption images are nearly equivalent to standard mammography, while providing two additional, complementary image modalities by means of phase and dark-field image contrast. The overall scan-time could be decreased to 12 seconds, which lies in between the typical scan times of 5–20 seconds reported for current tomosynthesis systems. Considering that state-of-art systems utilize X-ray anodes with a power output of up to 7 kW (200 mA, 35 kV), while the X-ray anode underlying the presented work was capped at 2.8 kW (70 mA, 40 kV), suggests that an additional reduction of exposure time and integrating detector noise is practicable. Within a clinical setting the mean glandular dose of 2.2 mGy could be even slightly decreased, by sparing one of the two polycarbonate-windows of the sample holder, using ultra-thin titan-wafers as substrate for the analyser grating and increasing the interferometer fringe visibility, which is currently only at 18%. Finally, we implemented an adaptable breast equivalent which allows the retrieval of artifact-free and quantitative images, hence providing comparability between subsequent breast measurements, indispensable for the diagnostic significance of future reader-studies.

To provide comparability between the clinical and experimental absorption images, the breast abladate was measured with an approximately doubled radiation dose in comparison to the conventional mammogram, contingent on the fact that 50% of all dose-relevant photons are attenuated by the analyser grating. Here the use of harder X-rays could facilitate a reduction of imaging dose, considering that phase-sensitive imaging offers strongly enhanced CNRs (in comparison to conventional absorption-based imaging) even at high energies [10–11]. Further, an optimal combination of the complementary, multimodal data may provide depiction of diagnostically crucial image content at much decreased imaging doses [12]. Finally, notice that the clinical absorption image (Fig 2E) is excelling the corresponding grating-based mammogram (Fig 2F), with respect to the depiction of the ultra-fine phantom structures #5, #6 and #10. However, a slightly inferior spatial resolution of the experimental vs. clinical scanner is understandable, when comparing underlying parameters: indirect X-ray conversion panel (Gadox) vs. direct X-ray conversion panel (amorphous selenium), one-dimensional anti-scatter-grid vs. two-dimensional anti-scatter-grid, and large focal spot (0.3mm) vs. small focal spot (0.05–0.2 mm) [13].

Due to a limited field-of-view, restricted by the size of the currently implemented detector grating, the final images presented in this paper were stitched from multiple projections and required a (undesired) scanning of the breast. However, recently the first gratings with a large field-of-view of 15 cm x 15 cm (round) and 30 cm x 5 cm have been produced. Besides, techniques for the fabrication of bended and tilted gratings, which are intended to adapt the cone-beam geometry of compact mammography systems in order to avoid beam shadowing, are rapidly developing.

Along with the clinically compatible imaging results presented here, we are convinced that in-vivo measurements are in near reach and that the development of a clinical prototype is now of highest priority.
